# SpectralNET – an application for spectral graph analysis and visualization

**DOI:** 10.1186/1471-2105-6-260

**Published:** 2005-10-19

**Authors:** Joshua J Forman, Paul A Clemons, Stuart L Schreiber, Stephen J Haggarty

**Affiliations:** 1 The Broad Institute of MIT & Harvard University, 320 Bent Street, Cambridge, MA 02141, USA; 2 Howard Hughes Medical Institute, Department of Chemistry & Chemical Biology, Harvard University, Cambridge, MA 02138, USA

## Abstract

**Background:**

Graph theory provides a computational framework for modeling a variety of datasets including those emerging from genomics, proteomics, and chemical genetics. Networks of genes, proteins, small molecules, or other objects of study can be represented as graphs of nodes (vertices) and interactions (edges) that can carry different weights. SpectralNET is a flexible application for analyzing and visualizing these biological and chemical networks.

**Results:**

Available both as a standalone .NET executable and as an ASP.NET web application, SpectralNET was designed specifically with the analysis of graph-theoretic metrics in mind, a computational task not easily accessible using currently available applications. Users can choose either to upload a network for analysis using a variety of input formats, or to have SpectralNET generate an idealized random network for comparison to a real-world dataset. Whichever graph-generation method is used, SpectralNET displays detailed information about each connected component of the graph, including graphs of degree distribution, clustering coefficient by degree, and average distance by degree. In addition, extensive information about the selected vertex is shown, including degree, clustering coefficient, various distance metrics, and the corresponding components of the adjacency, Laplacian, and normalized Laplacian eigenvectors. SpectralNET also displays several graph visualizations, including a linear dimensionality reduction for uploaded datasets (Principal Components Analysis) and a non-linear dimensionality reduction that provides an elegant view of global graph structure (Laplacian eigenvectors).

**Conclusion:**

SpectralNET provides an easily accessible means of analyzing graph-theoretic metrics for data modeling and dimensionality reduction. SpectralNET is publicly available as both a .NET application and an ASP.NET web application from . Source code is available upon request.

## Background

The field of graph theory concerns itself with the formal study of graphs – structures containing vertices and edges linking these vertices. Scientifically, graphs can be used to represent networks embodying many different relationships among data, including those emerging from genomics, proteomics, and chemical genetics. Networks of genes, proteins, small molecules, or other objects of study can be represented as nodes (vertices) and interactions (edges) that can carry different weights.

Graph-theoretic metrics, including eigenspectra, have been used to analyze diverse sets of data in the fields of computational chemistry and bioinformatics. Protein-protein interaction networks in *Saccharomyces cerevisiae*, for example, have been shown to exhibit scale-free properties [1], and databases of mRNAs can be mined using spectral properties of graphs created by their secondary structure [2]. Graph theory has also been used in conjunction with combinations of small-molecule probes to derive signatures of biological states using chemical-genomic profiling [3].

Despite the widespread use of graph theory in these fields, however, there are few user-friendly tools for analyzing network properties. SpectralNET is a graphical application that calculates a wide variety of graph-theoretic metrics, including eigenvalues and eigenvectors of the adjacency matrix (a simple matrix representation of the nodes and edges of a graph) [4], Laplacian matrix [5], and normalized Laplacian matrix, for networks that are either randomly generated or uploaded by the user. SpectralNET is available both as an ASP.NET web application and as a standalone .NET executable. While SpectralNET was originally written to analyze chemical genetic assay data, it should be of use to any researcher interested in graph-theoretic metrics and eigenspectra.

## Implementation

SpectralNET was originally written as an ASP.NET application in C#, and has subsequently been ported to a standalone .NET executable version (also written in C#). ASP.NET was originally chosen because it offered a fast, easy way to offer a thin client to users, obviating the need for large amounts of computational power on the client machine, as is often needed to perform large matrix calculations. A standalone version was created for three primary reasons: it avoids the problem of time-outs inherent when using a web interface (a potential issue when performing long-duration calculations), it is more easily distributable, and porting from ASP.NET to a .NET executable is a relatively simple matter.

Many computations are performed directly in C#, such as graph instantiation and metric calculation. Matrix computations (including eigendecomposition) are performed using the NMath Suite (CenterSpace Software, Corvallis, Oregon). Because the NMath Suite is a commercially licensed library, those receiving source code from the authors must supply their own means of performing matrix eigendecomposition in order to modify and redeploy the application. The implementation of the Fermi-Dirac integral, used in the calculation of spectral density, is ported from Michele Goano's implementation in FORTRAN (Goano, 1995). Because SpectralNET uses a third-party library for matrix calculations that is partially implemented using Managed Extensions for C++, SpectralNET will not be portable to Linux until the Mono implementation of this C++ language feature is complete.

## Results and discussion

### Graph creation

Idealized random networks can be automatically generated by the application, or networks can be uploaded by the user for analysis. SpectralNET can automatically generate random Erdos-Renyi graphs [7], Barabasi-Albert (scale-free) graphs [8], re-wiring Barabasi-Albert graphs [9], Watts-Strogatz (small-world) graphs [10], or hierarchical graphs [11]. Each automatically generated graph type is customizable with algorithmic parameters. SpectralNET was designed with extensibility in mind, so that users may request additional random graph types provided they submit a succinct algorithm to the author or create their own.

Networks can be uploaded by the user in the form of a Pajek file [12] or a tab-delimited text file with one edge per line (see additional file 1: HumanPPI_nodenodeweight.txt for an example network definition file defining a network of human protein-protein interactions). Raw data files can also be uploaded to the application, where each line of data is represented as a labeled vertex. Vertices can be connected with edge weights equal to the square of the correlation of their associated input data, or according to their Euclidean distance as defined by the Eigenmap algorithm [13]. If raw data is uploaded by the user, principal component analysis (PCA) [14,15] can optionally be performed on the data before calculating edge weights.

### Graph analysis

After processing the input network, SpectralNET displays for the user a wide variety of graph-analytic metrics. For example, the degree and clustering coefficient is displayed for each vertex. The degree of a vertex is the number of edges incident upon that vertex; for weighted graphs, SpectralNET calculates this as the sum of these edges' weights. The clustering coefficient of a node represents the proportion of its neighbors that are connected to each other, and is calculated for a node *i *as:

Ci(ki)=2niki(ki−1),     (1)
 MathType@MTEF@5@5@+=feaafiart1ev1aaatCvAUfKttLearuWrP9MDH5MBPbIqV92AaeXatLxBI9gBaebbnrfifHhDYfgasaacH8akY=wiFfYdH8Gipec8Eeeu0xXdbba9frFj0=OqFfea0dXdd9vqai=hGuQ8kuc9pgc9s8qqaq=dirpe0xb9q8qiLsFr0=vr0=vr0dc8meaabaqaciGacaGaaeqabaqabeGadaaakeaacqWGdbWqdaWgaaWcbaGaemyAaKgabeaakmaabmaabaGaem4AaS2aaSbaaSqaaiabdMgaPbqabaaakiaawIcacaGLPaaacqGH9aqpdaWcaaqaaiabikdaYiabd6gaUnaaBaaaleaacqWGPbqAaeqaaaGcbaGaem4AaS2aaSbaaSqaaiabdMgaPbqabaGcdaqadaqaaiabdUgaRnaaBaaaleaacqWGPbqAaeqaaOGaeyOeI0IaeGymaedacaGLOaGaayzkaaaaaiabcYcaSiaaxMaacaWLjaWaaeWaaeaacqaIXaqmaiaawIcacaGLPaaaaaa@46A8@

where *n*_*i *_denotes the number of edges connecting neighbors of node *i *to each other, and *k*_*i *_denotes the number of neighbors of node *i *[16]. In addition, the minimum, average, and maximum distances of each vertex are displayed, which are defined as the shortest, average, and maximum distances, respectively, from the node to any other node in the graph. The components of the adjacency, Laplacian, and normalized Laplacian eigenvectors corresponding to the vertex are also shown, where the adjacency matrix is defined as the matrix *A *with the following elements:

Aij(G)={1if i≠j and ∃ edge (i,j)0otherwise;                  (2)
 MathType@MTEF@5@5@+=feaafiart1ev1aaatCvAUfKttLearuWrP9MDH5MBPbIqV92AaeXatLxBI9gBaebbnrfifHhDYfgasaacH8akYBe9v8qqaqFD0xXdHaVhbbf9v8qqaqFr0xc9pk0xbba9q8WqFfeaY=biLkVcLq=JHqVepeea0=as0db9vqpepesP0xe9Fve9Fve9GapdbiqaaeGaciGaaiaabeqaaeqabiWaaaGcbaGaemyqae0aaSbaaSqaaiabdMgaPjabdQgaQbqabaGcdaqadaqaaiabdEeahbGaayjkaiaawMcaaiabg2da9maaceaabaqbaeaabiGaaaqaaiabigdaXaqaaiabbMgaPjabbAgaMjabbccaGiabdMgaPjabgcMi5kabbQgaQjabbccaGiabbggaHjabb6gaUjabbsgaKjabbccaGiabgoGiKiabbccaGiabbwgaLjabbsgaKjabbEgaNjabbwgaLjabbccaGmaabmaabaGaemyAaKMaeiilaWIaemOAaOgacaGLOaGaayzkaaaabaGaeGimaadabaGaem4Ba8MaemiDaqNaemiAaGMaemyzauMaemOCaiNaem4DaCNaemyAaKMaem4CamNaemyzaugaaaGaay5EaaGaei4oaSJaeiiOaaQaeiiOaaQaeiiOaaQaeiiOaaQaeiiOaaQaeiiOaaQaeiiOaaQaeiiOaaQaeiiOaaQaeiiOaaQaeiiOaaQaeiiOaaQaeiiOaaQaeiiOaaQaeiiOaaQaeiiOaaQaeiiOaaQaeiiOaaQaeiikaGIaeGOmaiJaeiykaKcaaa@8470@

the Laplacian matrix is defined as the matrix L with the following elements:

Lij(G)={diifi=j−w(e)ifi≠jand∃edgee(i,j)0otherwise,     (3)
 MathType@MTEF@5@5@+=feaafiart1ev1aaatCvAUfKttLearuWrP9MDH5MBPbIqV92AaeXatLxBI9gBaebbnrfifHhDYfgasaacH8akY=wiFfYdH8Gipec8Eeeu0xXdbba9frFj0=OqFfea0dXdd9vqai=hGuQ8kuc9pgc9s8qqaq=dirpe0xb9q8qiLsFr0=vr0=vr0dc8meaabaqaciGacaGaaeqabaqabeGadaaakeaacqWGmbatdaWgaaWcbaGaemyAaKMaemOAaOgabeaakiabcIcaOiabdEeahjabcMcaPiabg2da9maaceaabaqbaeaabmGaaaqaaiabdsgaKnaaBaaaleaacqWGPbqAaeqaaaGcbaacbiGae8xAaKMae8NzayMae8hiaaIae8hiaaIaemyAaKMaeyypa0JaemOAaOgabaGaeyOeI0Iaem4DaCNaeiikaGIaemyzauMaeiykaKcabaGaemyAaKMaemOzayMae8hiaaIae8hiaaIaemyAaKMaeyiyIKRaemOAaOMae8hiaaIae8hiaaIaemyyaeMaemOBa4MaemizaqMae8hiaaIae8hiaaIaey4aIqIae8hiaaIae8hiaaIaemyzauMaemizaqMaem4zaCMaemyzauMae8hiaaIae8hiaaIaemyzauMae8hiaaIae8hiaaYaaeWaaeaacqWGPbqAcqGGSaalcqWGQbGAaiaawIcacaGLPaaaaeaacqaIWaamaeaacqWGVbWBcqWG0baDcqWGObaAcqWGLbqzcqWGYbGCcqWG3bWDcqWGPbqAcqWGZbWCcqWGLbqzaaGaeiilaWIaaCzcaiaaxMaadaqadaqaaiabiodaZaGaayjkaiaawMcaaaGaay5Eaaaaaa@7915@

where *w*(*e*) denotes the weight of edge *e*; and the normalized Laplacian matrix is defined as the matrix L
 MathType@MTEF@5@5@+=feaafiart1ev1aaatCvAUfKttLearuWrP9MDH5MBPbIqV92AaeXatLxBI9gBamXvP5wqSXMqHnxAJn0BKvguHDwzZbqegm0B1jxALjhiov2DaebbnrfifHhDYfgasaacH8akY=wiFfYdH8Gipec8Eeeu0xXdbba9frFj0=OqFfea0dXdd9vqai=hGuQ8kuc9pgc9s8qqaq=dirpe0xb9q8qiLsFr0=vr0=vr0dc8meaabaqaciaacaGaaeqabaWaaeGaeaaakeaaimaacaWFmbaaaa@3967@ with the following elements:

Lij(G)={1if  i=j−1didjif  i  and  j  are  adjacent,0otherwise     (4)
 MathType@MTEF@5@5@+=feaafiart1ev1aaatCvAUfKttLearuWrP9MDH5MBPbIqV92AaeXatLxBI9gBamXvP5wqSXMqHnxAJn0BKvguHDwzZbqegm0B1jxALjhiov2DaebbnrfifHhDYfgasaacH8akY=wiFfYdH8Gipec8Eeeu0xXdbba9frFj0=OqFfea0dXdd9vqai=hGuQ8kuc9pgc9s8qqaq=dirpe0xb9q8qiLsFr0=vr0=vr0dc8meaabaqaciaacaGaaeqabaWaaeGaeaaakeaaimaacaWFmbWaaSbaaSqaaiabdMgaPjabdQgaQbqabaGccqGGOaakcqWGhbWrcqGGPaqkcqGH9aqpdaGabaqaauaabaqadiaaaeaacqaIXaqmaeaacqWGPbqAcqWGMbGzcaaMc8UaaGPaVlabdMgaPjabg2da9iabdQgaQbqaaiabgkHiTmaalaaabaGaeGymaedabaWaaOaaaeaacqWGKbazdaWgaaWcbaGaemyAaKgabeaakiabdsgaKnaaBaaaleaacqWGQbGAaeqaaaqabaaaaaGcbaGaemyAaKMaemOzayMaaGPaVlaaykW7cqWGPbqAcaaMc8UaaGPaVlabdggaHjabd6gaUjabdsgaKjaaykW7caaMc8UaemOAaOMaaGPaVlaaykW7cqWGHbqycqWGYbGCcqWGLbqzcaaMc8UaaGPaVlabdggaHjabdsgaKjabdQgaQjabdggaHjabdogaJjabdwgaLjabd6gaUjabdsha0jabcYcaSaqaaiabicdaWaqaaiabd+gaVjabdsha0jabdIgaOjabdwgaLjabdkhaYjabdEha3jabdMgaPjabdohaZjabdwgaLbaaaiaawUhaaiaaxMaacaWLjaGaeiikaGIaeGinaqJaeiykaKcaaa@8D64@

where *d*_*i *_denotes the degree of node *i *[5]. It should be noted that Chung defines the Laplacian matrix as the normalized form above, but we use the more commonly found definition (for an example, see Mohar [17]).

Many large networks derived from biological data are composed of multiple subgraphs that are not always connected together. SpectralNET computes many properties based on the selected or "active" connected component. For the active connected component, its size and average diameter are displayed in addition to graphs of degree distribution [18], clustering coefficient by degree, and average distance by degree [19]. Graphs of eigenvalues, eigenvectors, inverse participation ratios, and spectral densities of the three matrix types are also displayed. The inverse participation ratio is defined for each eigenvector as:

Ij=∑k=1N[(ej)k]4     (5)
 MathType@MTEF@5@5@+=feaafiart1ev1aaatCvAUfKttLearuWrP9MDH5MBPbIqV92AaeXatLxBI9gBaebbnrfifHhDYfgasaacH8akY=wiFfYdH8Gipec8Eeeu0xXdbba9frFj0=OqFfea0dXdd9vqai=hGuQ8kuc9pgc9s8qqaq=dirpe0xb9q8qiLsFr0=vr0=vr0dc8meaabaqaciGacaGaaeqabaqabeGadaaakeaacqWGjbqsdaWgaaWcbaGaemOAaOgabeaakiabg2da9maaqahabaWaamWaaeaadaqadaqaaiabdwgaLnaaBaaaleaacqWGQbGAaeqaaaGccaGLOaGaayzkaaWaaSbaaSqaaiabdUgaRbqabaaakiaawUfacaGLDbaadaahaaWcbeqaaiabisda0aaaaeaacqWGRbWAcqGH9aqpcqaIXaqmaeaacqWGobGta0GaeyyeIuoakiaaxMaacaWLjaWaaeWaaeaacqaI1aqnaiaawIcacaGLPaaaaaa@43FB@

where *e*_*j *_represents the eigenvector. Spectral density, or the density of the eigenvalues, is plotted for each eigenvalue as λ/Np(1−p)
 MathType@MTEF@5@5@+=feaafiart1ev1aaatCvAUfKttLearuWrP9MDH5MBPbIqV92AaeXatLxBI9gBaebbnrfifHhDYfgasaacH8akY=wiFfYdH8Gipec8Eeeu0xXdbba9frFj0=OqFfea0dXdd9vqai=hGuQ8kuc9pgc9s8qqaq=dirpe0xb9q8qiLsFr0=vr0=vr0dc8meaabaqaciGacaGaaeqabaqabeGadaaakeaacqaH7oaBcqGGVaWldaGcaaqaaiabd6eaojabdchaWnaabmaabaGaeGymaeJaeyOeI0IaemiCaahacaGLOaGaayzkaaaaleqaaaaa@36C0@ on the horizontal axis and pNp(1−p)
 MathType@MTEF@5@5@+=feaafiart1ev1aaatCvAUfKttLearuWrP9MDH5MBPbIqV92AaeXatLxBI9gBaebbnrfifHhDYfgasaacH8akY=wiFfYdH8Gipec8Eeeu0xXdbba9frFj0=OqFfea0dXdd9vqai=hGuQ8kuc9pgc9s8qqaq=dirpe0xb9q8qiLsFr0=vr0=vr0dc8meaabaqaciGacaGaaeqabaqabeGadaaakeaacqWGWbaCdaGcaaqaaiabd6eaojabdchaWjabcIcaOiabigdaXiabgkHiTiabdchaWjabcMcaPaWcbeaaaaa@35B8@ on the vertical axis, with the function p defined on any eigenvalue as:

p(λ)=1N∑j=1Nδ(λ−λj)     (6)
 MathType@MTEF@5@5@+=feaafiart1ev1aaatCvAUfKttLearuWrP9MDH5MBPbIqV92AaeXatLxBI9gBaebbnrfifHhDYfgasaacH8akY=wiFfYdH8Gipec8Eeeu0xXdbba9frFj0=OqFfea0dXdd9vqai=hGuQ8kuc9pgc9s8qqaq=dirpe0xb9q8qiLsFr0=vr0=vr0dc8meaabaqaciGacaGaaeqabaqabeGadaaakeaacqWGWbaCdaqadaqaaiabeU7aSbGaayjkaiaawMcaaiabg2da9maalaaabaGaeGymaedabaGaemOta4eaamaaqahabaGaeqiTdq2aaeWaaeaacqaH7oaBcqGHsislcqaH7oaBdaWgaaWcbaGaemOAaOgabeaaaOGaayjkaiaawMcaaaWcbaGaemOAaOMaeyypa0JaeGymaedabaGaemOta4eaniabggHiLdGccaWLjaGaaCzcamaabmaabaGaeGOnaydacaGLOaGaayzkaaaaaa@4820@

where *λ *is the eigenvalue and *δ *represents the delta function, implemented as described above [20]. Most graphs can be mouse-clicked to select the vertex corresponding to a desired data point, and eigenvalue graphs can be sorted by value or by vertex degree. All calculated graph metrics can be exported as a tab-delimited text file for further analysis.

### Visualization and dimensionality reduction

The main graph display window of SpectralNET offers two interactive graphical networks displays that support zooming and allow vertex selection by mouse-click. The default display view is the resulting graph processed by the Fruchterman-Reingold algorithm [21], which positions vertices by force-directed placement. The other available display is the network's Laplacian embedding, which locates vertices in two-dimensional Euclidean space using the corresponding second and third Laplacian eigenvector components (the first eigenvector component of the Laplacian matrix is degenerate). Exportation of the other Laplacian eigenvector components allows for visualization in higher dimensions.

In conjunction with uploaded raw data, Laplacian embedding allows the user to see a reduced-dimensionality view of high-dimensionality input, once this input is converted into a network. If the user chooses to process input data using the Eigenmap algorithm, Laplacian embedding shows the reduced-dimensionality result [13]. Dimensionality reduction has proven to be a useful tool in computational chemistry and bioinformatics; for example, Agrafiotis [22] used multidimensional scaling (MDS) to reduce the dimensionality of combinatorial library descriptors, and Lin [15] used PCA to analyze single nucleotide polymorphisms from genomic data. We chose to implement Laplacian embedding rather than MDS or other algorithms in SpectralNET because of promising results in the field of machine learning [23]. Although dimensionality reduction is especially useful for analyzing high-dimensional data, Laplacian embedding is an elegant display choice for any input network (see the next section for an example using a scale-free biological network). For a simpler (linear) dimensionality-reduced view of the input data, SpectralNET also has the option of viewing the results of PCA (though this view is not available when a network definition file, such as a Pajek file, is used). Both Laplacian embedding and PCA can be viewed in three dimensions with a Virtual Reality Modeling Language (VRML) viewer.

### Example analysis of a randomly-generated small-world network and a biological scale-free network

SpectralNET provides an easy-to-use interface for creating a randomly generated small-world network. All that is required is to supply the desired number of nodes, the desired number of neighbors to which to connect each node, and the desired random probability that an edge is re-wired. For this example we create a network with 300 nodes in which each node is connected to four neighbors, and edges are rewired with 4% probability.

**Figure 1:**
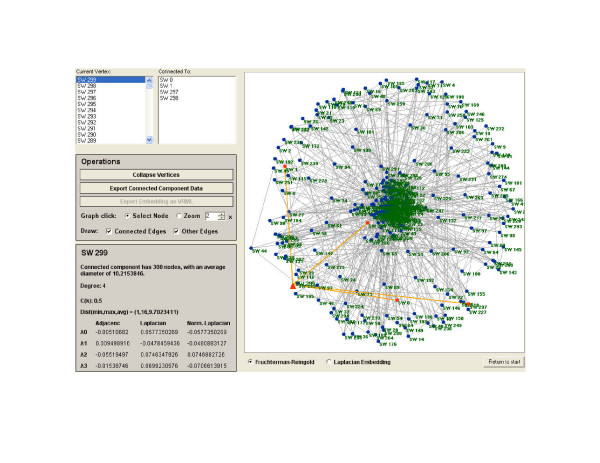
**Fruchterman-Reingold display of a small-world network**. Fruchterman-Reingold display of a randomly generated small-world graph. The node selection panel and node information panel are visible to the left of the display.

**Figure 2:**
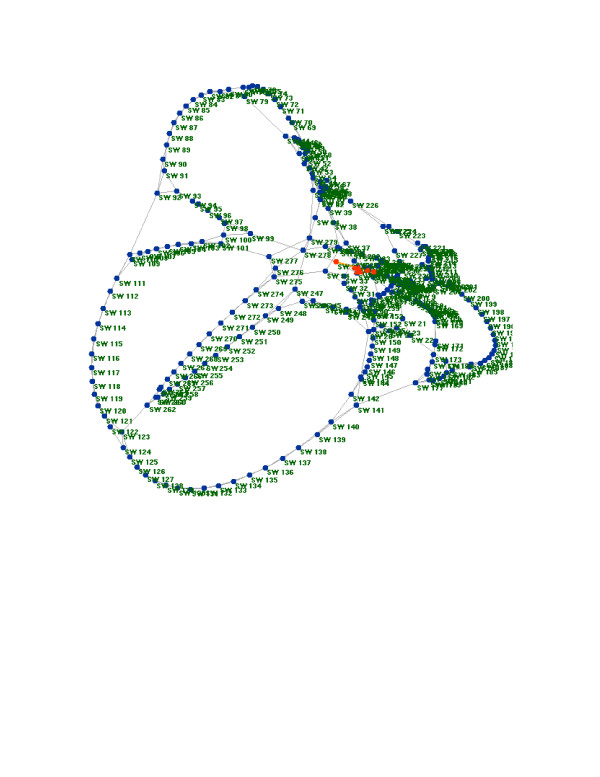
**Laplacian embedding of a small-world network**. Laplacian embedding of the randomly generated small-world network depicted in Figure 2, as drawn by SpectralNET.

The default view of the graph is its Fruchterman-Reingold display, which, as noted above, uses force-directed placement to draw graph nodes (Figure 1). While the Fruchterman-Reingold display offers a quickly generated view of large networks, relatively little information about the global organization of the network is observable in the display of this small-world network (one cannot tell, for example, that the graph is a small-world network by its Fruchterman-Reingold display alone). In order to see the graph as drawn by the Laplacian eigenvector components of each node, the "Laplacian Embedding" radio button underneath the graph display is selected. In contrast to the Fruchterman-Reingold display, the Laplacian embedding of this small-world network (Figure 2) conveys significantly more information about its topology. In this display, it is clear that the small-world network was generated by placing neighboring nodes next to each other in a ring-like fashion – the theoretical ring-structure is represented literally in the Laplacian embedding.

**Figure 3:**
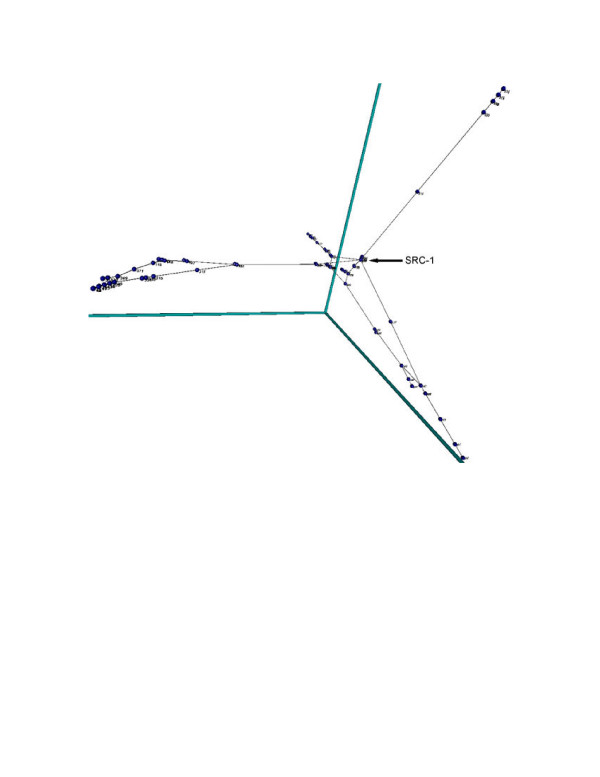
**Virtual Reality Modeling Language (VRML) diagram of a human protein interaction network**. Laplacian embedding of a scale-free biological network generated from a curated online database of protein interactions in humans (MIPS Mammalian Protein-Protein Interaction Database). For data see additional file 1 HumanPPI_nodenodeweight.txt.

Real-world biological networks are also amenable to topological analysis using Laplacian embeddings. In order to generate a suitable biological network to analyze, the MIPS Mammalian Protein-Protein Interaction Database [26] was downloaded and parsed into a node-node-weight file for import into SpectralNET (see: additional file 1: HumanPPI_nodenodeweight.txt). The Laplacian embedding of the largest connected component of the resulting graph (Figure 3) shows a central hub of highly connected proteins connected to four connected branches. Spectral analysis similar to that performed below shows that the network is scale-free in nature, as is further evidenced by the fact that there are many more low-degree proteins than high-degree proteins, with the relationship between number of proteins and protein degree following a power-law distribution (data not shown). The scale-free nature of this network suggests that highly-connected proteins in the central hub may perform a coordinating role for the proteins in this interaction network. Examining the most highly connected protein in the central hub of the network (indicated in Figure 3) shows that, indeed, it is the transcriptional co-activator SRC-1, which receives and augments signals from multiple pathways [27]. Readers with further interest in topological analysis of biological networks are encouraged to read Farkas et al. [28] for a global analysis of the transcriptional regulatory network of *S. cerevisiae *or Jeong et al. [29] for an analysis of the protein interaction network of the yeast.

**Figure 4:**
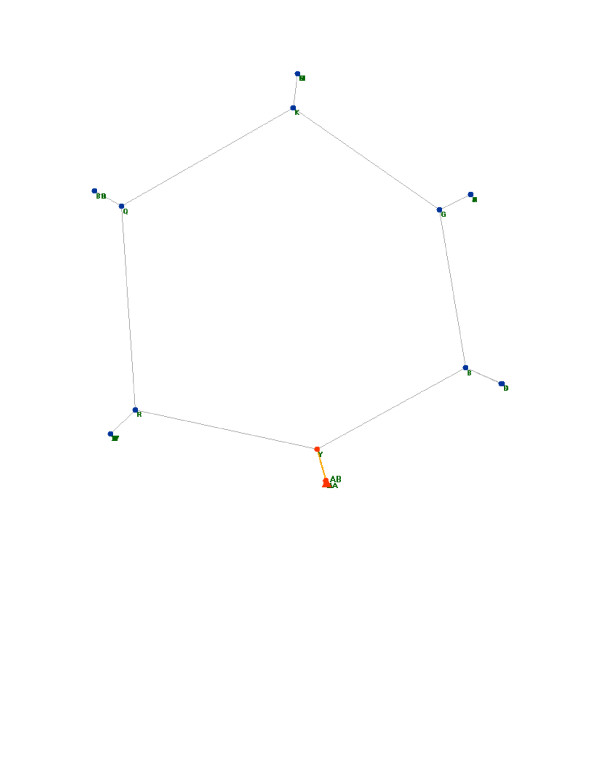
**Laplacian embedding of an uploaded small-world network**. Laplacian embedding of a small-world network (n = 33) created by attaching complete subgraphs to nodes arrayed in a ring. The subgraphs each appear as a single point because their constituent nodes have identical connectivity profiles, yielding identical Laplacian eigenvector components.

**Figure 5:**
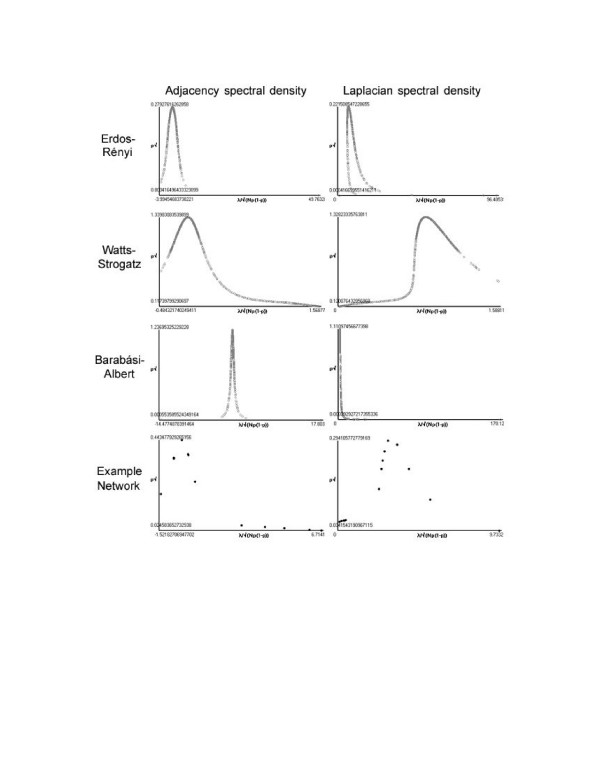
**Comparison of spectral properties of two small-world networks**. Plots of spectral density of the adjacency and Laplacian eigenvalues for a randomly-generated Erdos-Rényi graph, a randomly-generated Watts-Strogatz graph, a randomly-generated Barabási-Albert graph, and the small-world network depicted in Figure 4 consisting of complete subgraphs attached to nodes arrayed in a ring. The input small-world network is most similar to the randomly-generated Watts-Strogatz network, since they have the most similar topologies.

In addition to the graphical display of networks, SpectralNET enables analysis of spectral properties of input networks, which can shed light on graph topology. One way this can be achieved is to compare a small-world network similar to, but not identical to, the randomly generated small-world network described above. This graph is a small-world network created by attaching complete subgraphs, varying in size from three to six nodes, to nodes arrayed in a ring (see additional file 2: Small-world_nodenodeweight for the network definition file, originally described by Comellas [24]) (Figure 4). The spectral properties of this graph can be used to help identify the topology of the original graph, in this case by comparing their adjacency and Laplacian spectral densities (Figure 5) [5,20]. Spectral density measures the density of surrounding eigenvalues at each eigenvalue and serves as an especially useful metric of global graph topology. The plot of these values for the example network is most similar to the corresponding plots for a Watts-Strogatz network (in this network, there are 500 nodes connected to 6 neighbors, with a re-wiring probability of 1%), despite the fact that there are only 33 nodes in the example network. Thus, even when an example network has relatively few nodes, comparison of spectral properties of the graph to idealized graphs can yield clues about network's topology.

### Dimensionality reduction of a real-world chemical dataset to analyze QSAR

In addition to performing spectral analysis of networks, SpectralNET can also perform dimensionality reduction on chemical datasets to analyze quantitative structure activity relationships (QSAR). In this example, we upload a set of chemical descriptor data into SpectralNET and analyze it using the Laplacian Eigenmap algorithm originally developed by Belkin and Niyogi [13]. This dataset contains one small molecule, each created by the same diversity-oriented synthesis pathway [25], per row of the input file. Each column of the data represents a different *molecular descriptor *– metrics used to capture an aspect of the compound, such as volume, surface area, number of rings, etc.

The Laplacian Eigenmap algorithm in SpectralNET connects these small molecules to their *K*-nearest neighbors (measured by Euclidean distance), where *K *is an algorithmic parameter supplied by the user. In this example, we choose *K *= 7 to yield a reasonable number of edges in the resulting graph. Weights are assigned to each edge in one of two ways – every edge can have a weight of one, or weights can be assigned to edges by the following formula:

Wij=e−‖x1−x2‖2t     (7)
 MathType@MTEF@5@5@+=feaafiart1ev1aaatCvAUfKttLearuWrP9MDH5MBPbIqV92AaeXatLxBI9gBaebbnrfifHhDYfgasaacH8akY=wiFfYdH8Gipec8Eeeu0xXdbba9frFj0=OqFfea0dXdd9vqai=hGuQ8kuc9pgc9s8qqaq=dirpe0xb9q8qiLsFr0=vr0=vr0dc8meaabaqaciGacaGaaeqabaqabeGadaaakeaacqWGxbWvdaWgaaWcbaGaemyAaKMaemOAaOgabeaakiabg2da9iabdwgaLnaaCaaaleqabaGaeyOeI0YaaSaaaeaadaqbdaqaaiabdIha4naaBaaameaacqaIXaqmaeqaaSGaeyOeI0IaemiEaG3aaSbaaWqaaiabikdaYaqabaaaliaawMa7caGLkWoadaahaaadbeqaaiabikdaYaaaaSqaaiabdsha0baaaaGccaWLjaGaaCzcamaabmaabaGaeG4naCdacaGLOaGaayzkaaaaaa@441D@

where *W*_*ij *_represents the weight of an edge connecting edges *i *and *j *and *t *is an algorithmic parameter [13]. For the molecular descriptor dataset, edge weights of one were chosen (it should be noted that when applying the second method to this dataset, increasing values of *t *eventually resulted in convergence to the same result as this method around *t *= 20,000). SpectralNET also offers the choice of performing PCA on input data before performing the Laplacian Eigenmap algorithm, which is performed by default and remains enabled for this example.

**Figure 6:**
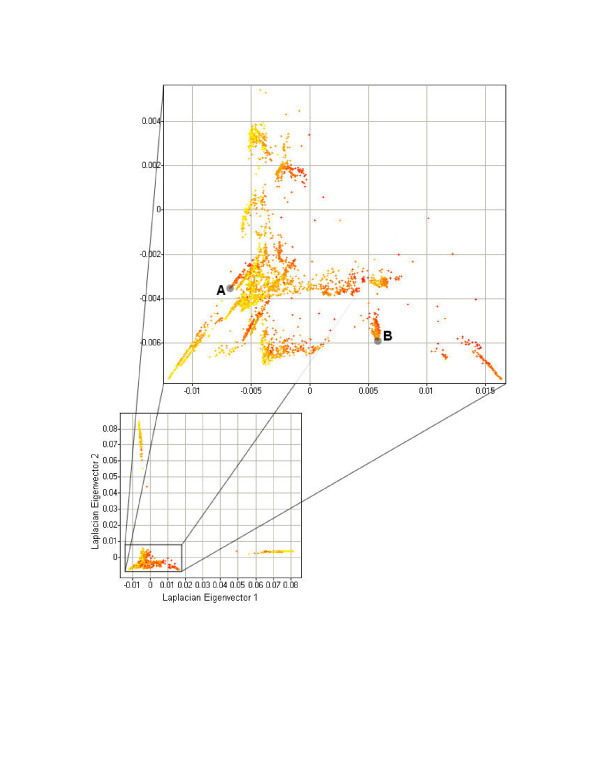
**Laplacian Eigenmap result for a molecular descriptor dataset**. A network of small molecules encoded as molecular descriptors, connected by similarity and displayed using the Laplacian Eigenmap algorithm, which plots each small molecule according to its corresponding Laplacian eigenvector components. Small molecules are colored according to the value of their minimized energy, one of the molecular descriptors of the original dataset.

The resultant Laplacian embedding of the graph, which can be viewed by selecting the "Laplacian Embedding" radio button underneath the graph view pane, is the reduced dimensionality result of the Laplacian Eigenmap algorithm (Figure 6). Like PCA, the Laplacian Eigenmap algorithm performs dimensionality reduction on an input dataset such that relationships among the data are captured by fewer dimensions. Unlike PCA, however, it is not a linear transformation of the data, and the resulting non-linear dimensionality reduction can offer a more powerful view of the data than does PCA.

**Figure 7:**
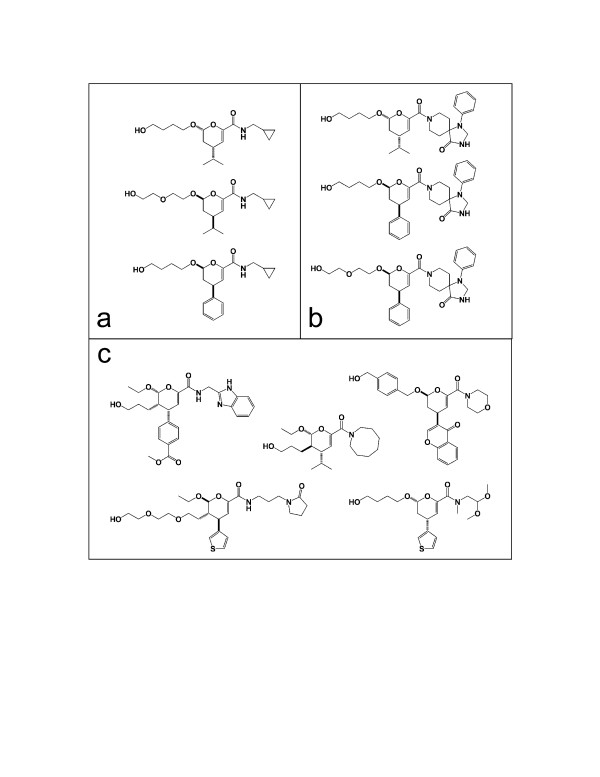
**Comparison of chemical structures from Laplacian Eigenmap clusters**. Comparison of chemical structures from the example real-world dataset of molecular descriptors depicted in Figure 5, taken either (A) from the group labeled "A", (B) from the group labeled "B", or (C) at random from the entire set.

**Figure 8:**
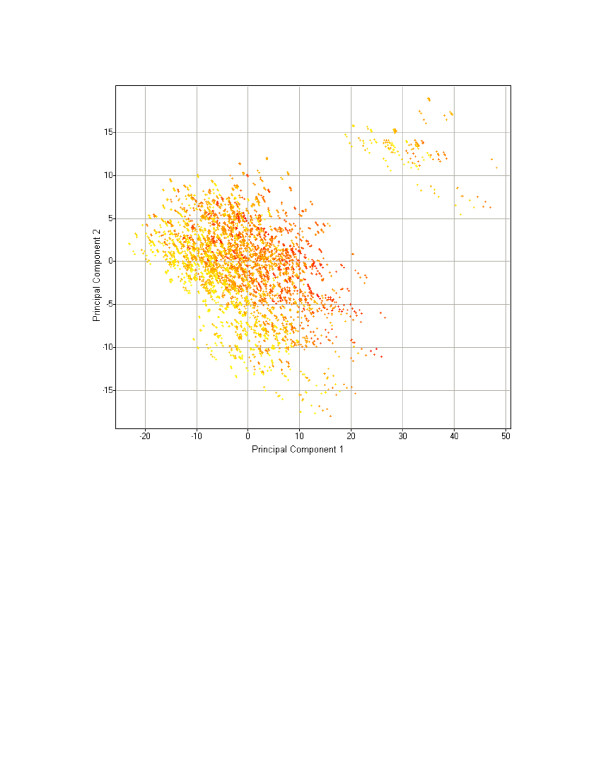
**Principal Components Analysis result for a molecular descriptor dataset**. The network of small molecules depicted in Figure 5, displayed using the first two principal components of the data as derived from PCA. Small molecules are colored according to the values of their minimized energies.

Because Laplacian Eigenmaps is a local, rather than global, algorithm, it seeks to preserve local topological features of the data in its reduced-dimensionality space [13]. Thus, it is difficult to compare its performance relative to a linear, global algorithm like PCA without labeled features on which to classify the data and a rigorous comparison across multiple datasets and datatypes. However, by visual inspection of points clustered together in the Laplacian Eigenmap result (from the highlighted areas in Figure 6), one can see that they are structurally similar relative to a set of random compounds selected from the space as a whole (Figure 7), and the two outlier groups visible in the original image are also chemically similar (data not shown). The same dataset plotted on its first two principal components (via PCA) yields no significant clustering comparable to that of Laplacian Eigenmaps with instead one large and a second smaller diffuse cluster visible (Figure 8). Additional support for nonlinear QSAR methods comes from Douali et al. [30], which found that a nonlinear QSAR approach using neural networks predicted activities very well, outperforming other methods found in the literature. A more rigorous comparison of these algorithms in the context of molecular descriptor data is ongoing.

## Conclusion

SpectralNET provides an easily accessible means of analyzing graph-theoretic metrics for data modeling and dimensionality reduction. The software allows users to analyze idealized random networks or uploaded real-world datasets, and exposes metrics like the clustering coefficient, average distance, and degree distribution in an easy-to-use graphical manner. In addition, SpectralNET calculates and plots eigenspectra for three important matrices related to the network and provides several powerful graph visualizations.

SpectralNET is available as both a standalone .NET executable and an ASP.NET web application. Source code is available by request from the author.

## Availability and requirements

**Project name: **SpectralNET

**Project home page: **

**Operating system(s): **Windows

**Programming language: **C#

**Other requirements: **The .NET framework v1.1 or higher

**License: **The SpectralNET software is provided "as is" with no guarantee or warranty of any kind. SpectralNET is freely redistributable in binary format for all non-commercial use. Source code is available to non-commercial users by request of the primary author. Any other use of the software requires special permission from the primary author.

**Any restriction to use by non-academics: **Contact authors

## Authors' contributions

JF developed and tested the software, wrote the initial version of the manuscript, and co-designed the software; PC provided feedback and data for molecular descriptor analysis, assisted with design of the software, and edited the manuscript; SS provided project guidance and edited the manuscript; SH initially conceived of and co-designed the software and edited the manuscript. All authors read and approved the final manuscript.

## Supplementary Material

Additional File 1 Human PPI network definition file. Network definition file representing a network of human protein-protein interactions. Data for this network was parsed from the MIPS Mammalian Protein-Protein Interaction Database. The numbers contained in this file correspond to the "shortLabel" annotation of proteins in the XML representation of the MIPS database. Click here for file

Additional File 2 Small-world network definition file. Network definition file for a 33-node small-world network with attached complete subgraphs. Click here for file
